# Antiviral Potential of Naphthoquinones Derivatives Encapsulated within Liposomes

**DOI:** 10.3390/molecules26216440

**Published:** 2021-10-25

**Authors:** Viveca Giongo, Annarita Falanga, Camilly P. Pires De Melo, Gustavo B. da Silva, Rosa Bellavita, Salvatore G. De-Simone, Izabel C. Paixão, Stefania Galdiero

**Affiliations:** 1 Programa de Pós-Graduação em Ciências e Biotecnologia, Department of Cellular and Molecular Biology, Biology Institute, Federal Fluminense University, Niterói 24020-141, Brazil; camillypestana@gmail.com (C.P.P.D.M.); dsimone@cdts.fiocruz.br (S.G.D.-S.); izabelpaixao@id.uff.br (I.C.P.); 2 Department of Agricultural Sciences, University of Naples Federico II, 80055 Portici, Italy; annarita.falanga@unina.it; 3 Department of of Fundamental Chemistry, Federal Rural University of Rio de Janeiro, Seropédica 23897-000, Brazil; gustavobezerrads@gmail.com; 4 Department of Pharmacy, University of Naples Federico II, 34102 Naples, Italy; rosa.bellavita@unina.it; 5 FIOCRUZ, Center for Technological Development in Health(CDTS)/National Institute of Science and Technology for Innovation in Neglected Diseases Populations (INCT-IDNP), Rio de Janeiro 21040-900, Brazil

**Keywords:** aminomethylnaphthoquinones, herpes simplex virus type 1, liposome drug carrier, nanoparticles

## Abstract

HSV infections, both type 1 and type 2, are among the most widespread viral diseases affecting people of all ages. Their symptoms could be mild, with cold sores up to 10 days of infection, blindness and encephalitis caused by HSV-1 affecting immunocompetent and immunosuppressed individuals. The severe effects derive from co-evolution with the host, resulting in immune evasion mechanisms, including latency and growing resistance to acyclovir and derivatives. An efficient alternative to controlling the spreading of HSV mutations is the exploitation of new drugs, and the possibility of enhancing their delivery through the encapsulation of drugs into nanoparticles, such as liposomes. In this work, liposomes were loaded with a series of 2-aminomethyl- 3-hydroxy-1,4-naphthoquinones derivatives with n-butyl (compound 1), benzyl (compound 2) and nitrobenzene (compound 3) substituents in the primary amine of naphthoquinone. They were previously identified to have significant inhibitory activity against HSV-1. All of the aminomethylnaphthoquinones derivatives encapsulated in the phosphatidylcholine liposomes were able to control the early and late phases of HSV-1 replication, especially those substituted with the benzyl (compound 2) and nitrobenzene (compound 3), which yields selective index values that are almost nine times more efficient than acyclovir. The growing interest of the industry in topical administration against HSV supports our choice of liposome as a drug carrier of aminomethylnaphthoquinones derivatives for formulations of in vivo pre-clinical assays.

## 1. Introduction

Approximately 67% of people under the age of 50 are infected with Herpes Simplex Virus 1 (HSV-1) and 13% of people aged 15–49 are infected with Herpes Simplex Virus 2 (HSV-2), urgently pushing the need for new therapies. Furthermore, in immunocompromised people, such as those with advanced HIV infection, HSV may have more severe symptoms and can also lead to more severe complications, such as encephalitis or keratitis [1,2]. HSV infections are efficiently treated with antiviral drugs, such as acyclovir (ACV) and its derivatives; however, long-term treatments may lead to drug resistance, mainly among immunocompromised patients, representing an additional critical emergence. Thus, there is an urgent need to explore new and effective strategies to face this problem. 

HSV-1 belongs to Alphaherpesvirinae sub-family responsible for the primary infection of epithelial cells, primarily followed by latency in neurons, and reactivation in the orolabial and genital mucosa throughout life. Effective treatments include the oral administration of valaciclovir and acyclovir for orolabial HSV, both in healthy and immunocompromised persons [3,4,5,6]. Mutations could explain the reactions against drug therapy on Thymidine Kinase and DNA polymerase, which results in the reduction or complete deficiency that impacts the efficacy of different anti-HSV drugs [7,8]. In addition, HSV-1 can control innate immunity by antagonizing tumor necrosis factor α [9] or through APOBEC3B and APOBEC3A and the degradation of tetherin [10,11].

Recent strategies to improve the biological activity of drugs against HSV-1 or HSV-2, and to overcome the issue of resistance, include the utilization of peptides [12,13,14,15,16,17] and nanotechnology as delivery strategies for injured tissues [12,13,14,15,18,19,20,21,22,23].

Liposomes are artificially produced vesicles that are formed by layers of natural or synthetic phospholipids, widely exploited for diagnosis, vaccines, and delivery of nutrients and bioactive molecules. Their bioavailability and reduced toxic effects make liposomes the most widely used carriers among nanoparticles [24,25]. As drug delivery tools, they can transport both hydrophilic substances that are localized in the liquid phase of the vesicles and hydrophobic molecules immersed in the phospholipid bilayer [26,27]. 

Another important strategy in antiviral drug development research is based on natural compounds and the chemical synthesis of analogues of natural products, such as lawsone (2-hydroxy-1,4-naphthoquinone), which provide unlimited availability and chemical diversity. In this sense, naphthoquinone derivatives are widely recognized as potent antiviral, antitumoral, and antiparasitic molecules, the effects of which include apoptosis, proteasome inhibition and anti-inflammatory process control, through a reduction in INOS expression [28,29,30,31,32,33,34,35].

In previous work, we demonstrated that Mannich base, derived from lawsone, has the ability to control herpesvirus replication in vitro [36,37,38]. Although most studies reveal that lawsone is effective mainly on tumoral cells, we showed that aminomethylnaphthoquinone derivatives could inhibit both the early and late phases of replication in two different models of the Herpesviridae family: Herpes Bovine type 5 and Herpes Simplex type 1 [36,37]. Moreover, in BALB/c models, aminomethylnaphthoquinones, with a butyl substituent, have low toxicity and could be a good candidate against HSV-1 [37]. 

To obtain an effective anti-HSV-1 drug, the development of a delivery strategy to enhance internalization, reduce the administration dose and, consequently, undesired side effects is necessary. Here, we prepared liposomes encapsulating these aminomethylnaphthoquinone derivatives to be analyzed in pre- and post-treatment antiherpetic assays. To date, there are no reports in the literature on the antiviral activity of this class of compounds encapsulated into liposomes. Our results suggest that ACV and naphthoquinones, delivered through a liposomal system, may improve clinical efficacy and decrease adverse effects, such as toxicity.

## 2. Results

The drugs used in this study are hydrophobic, and thus their application is somewhat impaired. For this reason, the use of carriers is a widely accepted strategy to improve their delivery. Liposomes represent an attractive approach to achieve this objective, since it is possible to encapsulate both hydrophilic molecules in the core or hydrophobic molecules in the bilayer. 

**Figure 1:**
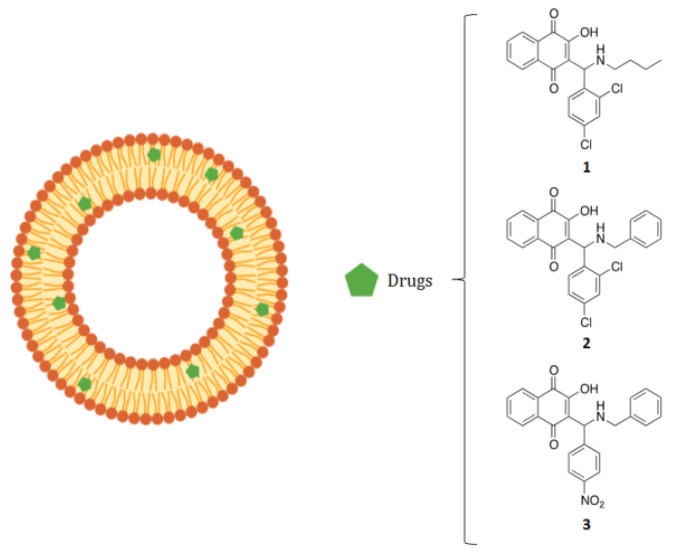
Schematic representation of liposomes loaded with a series of 2-aminomethyl-3 hydroxy 1,4 naphthoquinones derivatives (1 to 3), used in this study to determine anti-HSV activity.

We prepared liposomes encapsulating three hydrophobic molecules that were purified by gel filtration to separate the non-encapsulated drugs. Our data clearly indicate that the drugs are completely encapsulated in the experimental conditions used in this study. Moreover, the ratio between the drug and the lipid concentrations is 0.01, indicating that the encapsulated drug is at a much lower concentration, which is not able to influence liposome packing. Figure 1 presents a scheme of the drug, encapsulated inside liposomes and the chemical structures of the three drugs.

Liposomes loaded with drugs were characterized by DLS. Table 1 presents their hydrodynamic diameters, which vary from ca. 102 to 130 nm with a polydispersity index of lower than 0.2, indicating that monodispersed preparation is suitable for applications in biomedicine. Furthermore, the surface charge of the liposomes, as determined by the measurement of their zeta potential (Table 1) showed values of between −13 and −24, indicating the colloidal stability of the prepared formulations.

At 24 and 48 h, the release of the drug from the liposomes was observed. Our data clearly show that the concentration of compounds encapsulated in the liposomes remained stable for up to 48 h. The dimensions of the liposomes were also checked after 48 h, with no significant change.

Initially, we evaluated the influence of liposomes on cell viability. For this, monolayers of Vero cells (10^4^ cells/mL) were incubated with several concentrations of 2-aminomethyl-3-hydroxy-1,4 naphthoquinone derivatives that either were or were not encapsulated in the liposome (0.5 to 10 μM) for 48 h at 37 °C (Table 2). Following this, 1 mg/mL of 3-(4,5-Dimethylthiazol-2-yl)-2,5 Diphenyl Tetrazolium Bromide was added to each well for 4 h at 37 °C and the resulting purple formazan was followed at 570 nm. Since liposomes are considered an excellent delivery system, it may be reasonable for the liposome to increase the cytotoxic effect on cells. The CC_50_ values showed that all derivatives could be considered more toxic in the presence of Egg-phosphocholine (Table 2), but for all of the experiments, the maximum concentration used was below that of the CC_50_ values.

Our results also highlighted the influence of the substituent on the values of CC_50_. The presence of benzyl in the primary amine of naphthoquinone derivatives influenced the compound 2 value (11 ± 1 μ), which was shown to be the most toxic among all of the derivatives. Both compound 3, with the nitrobenzene substituent, and acyclovir present the same CC_50_ values (13 ± 2 and 13 ± 1 μM, respectively), while the presence of a butyl radical in compound 1 was determined to have minimal harmful effects on Vero cells (15 ± 1 μM).

**Figure 2:**
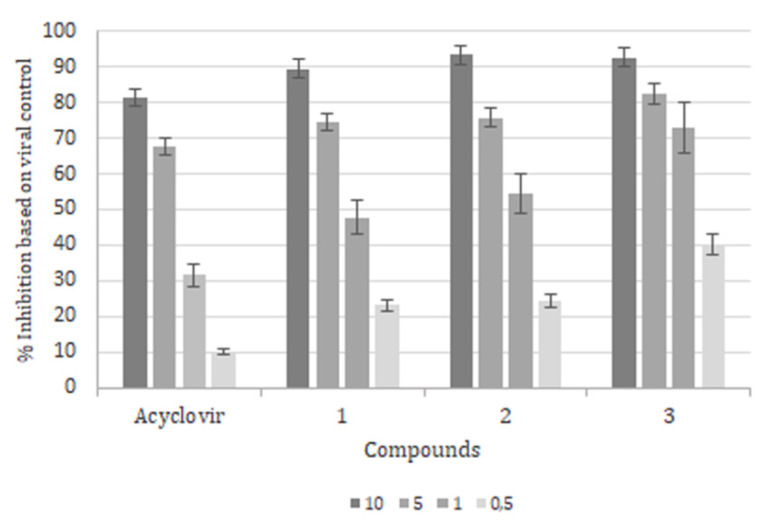
Effects of 2-aminomethyl-3-hydroxy-1,4 naphthoquinones encapsulated in liposomes on HSV-1 replication. After infection (MOI = 0.1) Vero cells (3 × 10^5^ cells/well) were grown in the presence of 0.01 to 10 µM of compounds 1–3 for 24 h. Inhibition was calculated based on plaque-forming units of viral control. The results were expressed as the Mean ± SD of three independent experiments. *p* < 0.05 control group.

To verify if the encapsulated compounds could also inhibit HSV-1 replication, we performed a yield-reduction assay (Figure 2). Briefly, after incubation with HSV-1 (MOI of 0.1) for 1 h at 37 °C, cells were washed with MEM 5% FCS and incubated with acyclovir, or each of the aminomethylnaphthoquinone derivatives encapsulated in liposomes at concentrations ranging from 0.01 to 10 μM for 24 h in atmosphere, with 5% CO_2_ at 37 °C. After the dilution (1:10) of the viral suspension, new 24-well plates were used to determine the EC_50_ values, based on viral control. EC_50_ is a measure of the inhibition of viral replication in the presence of several drug concentrations, and the lowest is the EC_50_ value; the most effective is the drug which controls in vitro replication. 

All of the encapsulated 2-aminomethyl-3-hydroxy-1,4 naphthoquinone derivatives exhibited lower EC_50_, compared to the positive control acyclovir (see Table 3). The presence of nitrobenzene (compound 3) and benzyl (compound 2) substituents in the aminomethylnaphthoquinone structures conferred the best results for viral inhibition with 0.36 ± 0.04 μM and 0.56 ± 0.02 μM, respectively, and almost four and nine times the activity of acyclovir in the same conditions (3.16 ± 0.09 μM). Even compound 1, with the lowest antiviral activity among derivatives (1.73 ± 0.08 μM), showed that the butyl substituent was more effective than acyclovir in inhibiting HSV-1 replication (Table 3). 

In terms of toxicity and antiviral effect, the selective index (SI), calculated through the CC_50_/EC_50_ ratio, represents how promising the candidate is for further in vitro and in vivo studies. First, our results showed that all encapsulated compounds presented higher SI values compared to acyclovir (SI = 4.1) (Table 3). In fact, the relationship between CC_50_ and EC_50_ represents the lowest value of this series (SI = 8.7 μM) for the n-butyl derivative (compound 1), but still almost twice that of the control; in particular, although being less toxic, compound 1 had the highest EC_50_ value. Among all derivatives, the most relevant antiviral activity was obtained with the nitrobenzene radical (compound 3) (SI = 36), mainly due the significant reduction in drug concentration to the EC_50_ (0.36 ± 0.04), followed by compound 2 (with benzyl radical) (SI value of 20), which also had significant biological activity.

In comparison, the inhibitory effect of non-encapsulated derivatives was clearly observed in compound 1 (butyl) and compound 2 (benzyl), with the most effective SI values (1.52 and 1.16, respectively, data not shown). In concentrations of up to 10 μM, the CC_50_/EC_50_ ratio provides compound 2 (benzyl) with the highest SI value (20.75), mainly due the lower toxicity (CC_50_ = 22.0 ± 1.6 μM); the highest antiviral effects (EC_50_ = 1.06 ± 0.49 μM) were observed in compound 1 with n-butyl—SI = 9.6 (CC_50_ = 19 ± 1.52 μM and EC_50_ = 1.98 ± 0.3 μM)—and compound 3 with nitrobenzene—SI value of 5.48 (CC50 = 17.0 ± 2.0 μM; EC50 = 3.1 + 0.18 μM).

The comparison of the SI values with free and encapsulated derivatives showed that liposomes, as carriers, enhanced the antiviral effect of these compounds, even with discreet toxicity. 

**Figure 3:**
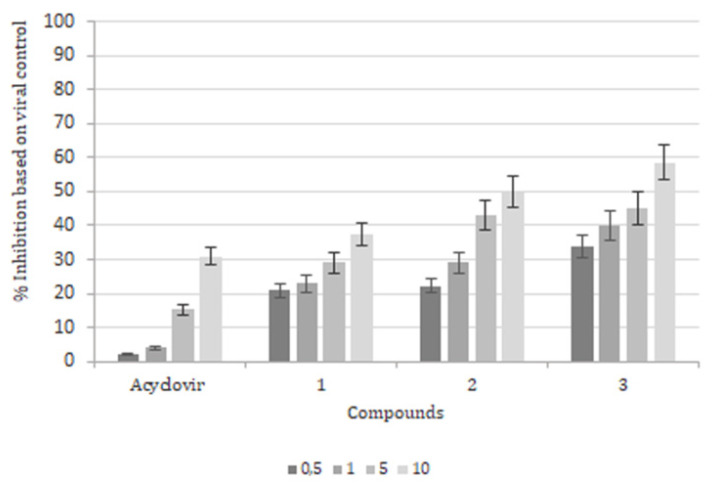
Attachment assay. Vero cells (3 × 10^5^ cells/well) were incubated for 2 h with HSV (MOI = 0.1) at 4 °C in the presence or absence of 2-aminomethyl-3-hydroxy-1,4 naphthoquinones encapsulated into liposomes. The level of infection was determined 48 h later by plaque-forming unit counts. The results were expressed as Mean ± SD of three independent experiments. *p* < 0.05 control group.

We performed a series of attachment and time-addition assays. First, the infected Vero cells, with HSV-1 (MOI of 0.1) in the presence of 2-aminomethyl-3-hydroxy-1,4 naphthoquinone derivatives and acyclovir, were encapsulated in liposomes for 2 h at 4 °C. Following this, the cells were washed twice with ice-cold PBS and covered with 5% MEM and 2% methylcellulose for 48h at 37 °C. The virus-binding assay demonstrated moderate activity for all compounds and acyclovir. The maximum inhibition did not exceed 58.3% with compound 3 (nitrobenzene) and 49.7% with compound 2 (benzyl) at 10 μM. However, the n-butyl substituent (compound 1) had the lowest inhibition value (37.6%), but this was still higher compared to the 30.5% of acyclovir (Figure 3).

**Figure 4:**
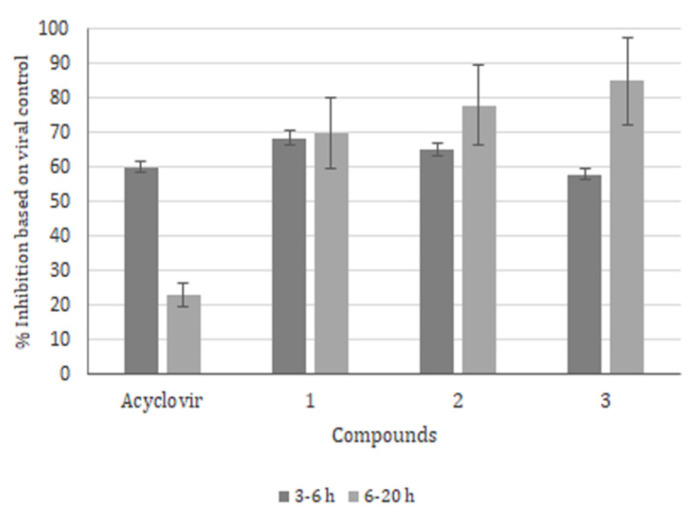
Time of addition assay. Vero cells were first incubated with HSV-1 (MOI = 0.1) for 1 h, then acyclovir (12.6 μM), compound 1 (6.92 μM), 2 (2.24 μM) and 3 (1.44 μM) were added at different incubation times, as indicated. The level of infection was determined 48 h later by plaque-forming unit counts. The results are expressed as Mean ± SD of three independent experiments. *p* < 0.05 control group.

The time of addition assay is a common approach for determining how long the addition of a specific compound could remain efficient for controlling viral replication in cell culture. For this purpose, in order to compare if liposomes were also able to inhibit the early and late phases of HSV-1 replication, we used protocols, already published by our group, with free derivatives [38]. Briefly, after initial HSV-1 infection with 0.1 MOI, Vero cells were washed with PBS and incubated with MEM 5%BFS for 3 h post infection (hpi) or 6 hpi at 37 °C. Subsequently, the medium was replaced by naphthoquinone derivatives, and acyclovir was encapsulated into liposomes with concentrations corresponding to four times the EC_50_ values for an additional 3 h or 14 h of incubation. Our results showed that all compounds were effective in blocking the early phase (3–6 hpi) of HSV-1 replication (Figure 4). Compounds 1 (n-butyl radical) and 2 (benzyl radical) showed very similar inhibition values (69% and 65%, respectively), while compound 3 was the least efficient (58%) in terms of controlling the early phase of HSV-1 replication, probably targeting the essential components of virus replication, such as polymerase, thymidine kinase and the helicase-primase (58%).

Moreover, the efficacy of compound 3 was evident in the late phase (85%), proving to be more active than all aminomethylnaphthoquinones; however, this tendency was also observed for compound 1 (70%) and compound 2 (78%), indicating that all series act as blockers of both phases (Figure 4). In fact, the most effective was compound 3, with a significant SI value (36), having equal the ability to keep the cells alive while blocking some of the still-unknown targets of HSV-1 replication.

## 3. Discussion and Conclusions

Over the last few decades, anti-HSV-1 drug development has essentially been based on the modification of the acyclovir prototype; as a matter of fact, currently, the three classes of licensed HSV-1 drugs act on viral DNA replication. The typical use of acyclovir and penciclovir often presents limitations due to their side effects and low efficacy, determined by drug-resistant strains. Viral resistance to ACV has been shown to be more common in immunocompromised patients undergoing long-term therapy, as seen for most other viral infections, highlighting the need for new drugs with novel mechanisms of action [39,40]. 

In recent years, the literature has been filled with scientific reports of natural and synthetic compounds with anti-herpetic activities [24,41,42,43]; unfortunately, most present a significant level of toxicity. In this sense, liposomes act as efficient vehicles, significantly reducing the dose of the drug being administered and, thus, its toxic level, as demonstrated for liposomal formulations containing doxorubicin [44] and amphotericin [45]. 

This work evaluated the antiviral activities of the naphthoquinone derivative encapsulated into liposomes and compared them with those of free drugs. Naphthoquinones are natural compounds that are widely found in plants, microorganisms, and animals with significant biological activities (anti-inflammatory, anti-microbial and cytotoxic) against cancer. Inside cells, they produce stable free radicals, inducing oxidative stress and caspase 3/7 activity, and irreversibly complex proteins, generally leading to the inactivation and loss of protein function in many types of cells [28]. Although natural and synthetic, naphthoquinones have been extensively studied as anticancer drugs. Some derivatives, such as lawsone and 2-aminomethyl-3-hydroxy-1,4 naphthoquinones have also been shown to have antiviral activities against bovine herpesvirus (BoHV-5) and HSV-1 [36,38]. 

Previous studies with 2-aminomethyl-3 hydroxy 1,4 naphthoquinones, carrying butyl and benzyl substitutions, found that they were the most promising compound against HSV-1, with SI values of 1.52 and 1.16, respectively, which are higher than ACV (SI = 0.80). The same effectiveness was observed with nitrobenzene derivative in the inhibition of BoHV-5 replication, demonstrating possibly different targets in the same viral family, despite the same control on the early and late phases of replication [36,38] Furthermore, pre-clinical studies with BALB/c demonstrated that the oral administration of compound 1 (butyl) has no effect on transaminases level or kidneys functions, excluding possible side effects after the oral administration of the substance [37]. 

These compounds were encapsulated in neutral PC liposomes to verify the possible differences in biological activities of aminonaphthoquinones, with concentrations up to 10 μM. The hypothesis is that liposomes could improve biological activity, enhancing solubilization and reducing administration dose. Our viability results demonstrate a discrete reduction in CC_50_ values of the 2-aminomethyl-3hydroxy-1,4 naphthoquinones in the presence of liposomes. Nevertheless, antiviral activity improves. 

The first antiviral assay demonstrated several differences, which are likely based on substitutions in the amino or naphthoquinone structure. The presence of substitutions provided an antiviral effect higher than drug control (acyclovir). In particular, nitrobenzene derivate (compound 3) gives the highest antiviral effect with a value of 0.36 μM. However, the EC_50_ differences between compounds 2 and 3 were not significant compared to acyclovir. The selective index (SI) calculated by the ratio between cytotoxic and antiviral values gives to compound 3 the most effective antiviral effect (SI = 36) and almost nine times the value obtained for acyclovir (SI = 4). Comparing the biological parameters concerning compound 2, with substituted benzene, we found that there was both a reduction in cell viability and antiviral effects with an SI value of 20. The benzyl substitution confers to compound 2 a higher activity compared to compound 1 (SI = 8.7). our results using free compounds with concentrations up to 10 μM showed that for n-butyl substituted derivative, the same EC_50_ value showed when encapsulated into liposomes (1.73 μM). [38]. However, the different SI value for the encapsulated drug could reduce the toxic effect, which is important mainly in prolonged therapies. The other two derivatives, benzyl and substituted nitrobenzene, being more hydrophobic than compound 1, showed an enhancement in their antiviral effect when delivered through liposomes. It is likely that, when located in the bilayer of the liposomes, they are more easily delivered inside the Vero cells, where they can exert their activity. It is interesting to note that, in our previous study, the benzyl-substituted derivative was the most effective antiviral compound in the series, but when encapsulated into liposomes, it was replaced by compound 3, showing that the aliphatic group of compound 1 in the liposome structure affected the available concentration of this derivative to the cell. The formulation containing the acyclovir required higher concentration for activity, compared to the free molecule. That being said, it still undoubtedly presents an advantage, as the SI value was increased by more than five times (4.1 μM) compared to free acyclovir (0.80 μM). In conclusion, for the first time, we demonstrated that liposomes can equally be considered a suitable carrier for acyclovir and naphthoquinones derivatives. 

To identify the possible targets of encapsulated drugs, we choose specific points in HSV-1 replication. First the virion attaches to the cell membrane and, after penetration, the early and late phases of HSV-1 replication. These experiments allowed us to compare the results with those previously obtained for the free derivatives.

Viral inhibition during the attachment phase was not efficient with liposomes, reaching a maximum value of 58% with compound 3 and 50% with compound 2 at 10 μM. The lowest activity was obtained with the n-butyl substituent (38%), but we showed that all derivative formulations were still more effective than acyclovir (30%).

The use of four times the EC_50_ values at the time of the addition assay showed that all compounds were more effective than acyclovir in controlling viral infection during the early and late phases of replication. At the same time, the importance of incubation in the early phase represents the possible blockage of proteins involved in viral DNA replication, such as the viral polymerase and thymidine kinase of HSV, the latest tagged in the maturation and budding of the virion from the nucleus of the infected cell. The nitrobenzene-substituted derivatives revealed the lowest activity during the first 3 and 6 h post infection, but butyl- and benzene-substituted molecules also present very similar inhibition percentages that were considered to be not so effective, compared to that shown in the late phase. Accordingly, our previous results revealed that these naphthoquinone derivatives could interact with the proteins responsible for organizing the viral nucleocapsid, and this fact supports our objective of searching for a new target other than the thymidine kinase of HSV-1. The efficacy of compound 3 was evident (85%), followed by compound 1 (70%) and compound 2 (78%). We conclude this preliminary study with the observation that the neutral liposome could carry anti-HSV-1 compounds of naphthoquinone origin, and further studies are necessary to enhance internalization and unravel the mechanism of their activity.

## 4. Materials and Methods

### 4.1. Compounds 

Three molecules of 2-aminomethyl-3-hydroxy-1,4-naphthoquinones 1–3 (Figure 1) were synthesized as Mannich bases with some modifications [46]. Their identities were confirmed by ^1^H NMR (Varian VNMRS 300 MHz spectrometer) and their purity determined by elemental analysis (Perkin-Elmer CHN 2400 micro analyzer at Central Analítica IQ-USP, SP, Brazil) and melting point measurements (Digital Melting Point IA9100, ThermoFischer Scientific, Waltham, MA, USA). They were dissolved in dimethyl sulfoxide (DMSO), 100% sterile, and stored at −20 °C. The stock solutions (50 mM) were diluted in MEM (Sigma-Aldrich) for the tests. 

### 4.2. Cell Culture and Virus

Vero cells (ATCC CCL-81) from African green monkey kidney cells (Cercopithecus aethiops) were cultured in Minimum Essential Medium (MEM) (Sigma-Aldrich, St. Louis, MO, USA.), supplemented with 5% FBS (HyClone, Logan, UT, USA), 100 U/mL penicillin and 100 mg/mL streptomycin at 37 °C in 5% CO_2_ atmosphere. For all antiviral tests, HSV-1 strain SC-16 (ATCC) and fibroblast cells at 80% confluence were used.

### 4.3. Liposome Preparation

Lipid stock solutions of egg phosphatidylcholine (PC) (Avanti Polar Lipid Inc., Alabaster, AL, USA) (0.1 mM) were prepared in chloroform, containing 30% vol. methanol. Mixtures of appropriate amounts of PC and aminomethylnaphthoquinones (0.5 to 10 mM) were prepared, and the organic solvent was evaporated under a gentle stream of nitrogen. Following this, lipid films were kept in a vacuum overnight to remove the residual organic solvent and hydrated with PBS buffer at pH 7.4 for 1 h. The lipid suspension was freeze–thawed 6 times, LUVs were passed for 10 cycles through a 100 nm pore size according to the extrusion method [47] (LipexTM, Avanti Polar Lipid Inc., Alabaster, AL, USA). Unloaded drugs were removed by the Sephadex G50 column to purify the final formulation and evaluate the efficiency of encapsulation. Dynamic light scattering (DLS) measurements were made using Zetasizer Nano-ZS (Malvern Instruments, Worcestershire, UK), to check the Zeta potential, size, expressed as z-average, and polydispersity index (PDI) of the loaded liposomes (Table 1).

### 4.4. In Vitro Drug Release

The in vitro drug release from liposomes was determined using UV–vis spectrophotometry. Liposomes encapsulating the drug were dialyzed against water under continuous stirring at 37 °C; 100 mL aliquots were withdrawn at 24 and 48 h and replaced with an equal volume of fresh water. The free drug was quantified based on the UV– vis absorbance at 260 nm, using a previously established calibration curve.

### 4.5. Cytotoxicity Assay 

Vero cells cultivated in 96-multiwell plates (1 × 10^4^ cells/well) were incubated with liposomes coupled to 2-aminomethyl-3-hydroxy-1,4-naphthoquinones in different concentrations (0.5, 1, 5 and 10 μM) for 48 h at 37 °C and 5% CO_2_ atmosphere. Then, 50 μL of 3-(4,5-dimethylthiazol-2-yl)-2,5 diphenyl MTT (1 mg/mL, Sigma-Aldrich, St Louis, MO, USA) was added to each well for 4 h at 37 °C [48]. The MTT reduction in living cells creates formazan, a purple compound that is absorbed at 570 nm. The 50% cytotoxic concentration (CC_50_) was calculated by linear regression analysis of the dose–response curves.

### 4.6. Antiviral Assays 

For all antiviral assays, strain SC-16 HSV-1 was used at a multiplicity of infection (MOI) of 0.1 to infect Vero cells at 3 × 10^5^ cells/well using a modified yield reduction assay [49]. All aminomethylnaphthoquinone derivatives were previously diluted in pre-chilled MEM with 5% FCS.

#### 4.6.1. Yield Reduction Assay

To determine the HSV-1 title, Vero cells maintained in 24-multiwell plates (3 × 10^5^ cell/well) were infected with HSV-1 strain SC-16 (MOI of 1) for 1 h at 37 °C and 5% CO_2_ atmosphere.

After the removal of the viral inoculum, cells were treated with 0.01 µM to 10 µM of compound 1, 2, 3 and ACV encapsulated in liposomes for 24 h at 37 °C and 5% CO_2_ atmosphere. Then, the cells were subjected to three cycles of freezing and thawing and the inoculum diluted (1:10) to a new infection in 24-multiwell plates (10^5^ cells/well) for 1 h at 37 ºC and 5% CO_2_ atmosphere. The cells were covered with MEM 2X, 5% FCS and 2% methylcellulose for 48h at 37 °C and the viral title was determined by the number of viral plaque units per mL (PFU/mL). EC_50_ values, which means the drug concentration able to inhibit 50% of the viral plaque formation, were determined by linear regression compared to the untreated infected control.

#### 4.6.2. Attachment Assay

A virus-binding assay was performed with pre-chilled Vero cells at 4 °C for 1 h in 24-well plates (3 × 10^5^ cell/well). The medium was removed, and the monolayers were inoculated with HSV-1 (0.1 PFU/cell) in the presence of 0.5 µM, 1 µM, 5 µM and 10 µM of compound 1, 2, 3 or ACV with liposomes for 2 h at 4 °C. Then, cells were washed three times with iced PBS and covered with MEM 2X, 5% of fetal bovine serum and 2% methylcellulose for 48 h at 37 °C. The number of viral plaque units per mL (PFU/mL) was calculated, corresponding to inhibition based on viral control. 

#### 4.6.3. Time-of-addition Assay

To verify if the series of 2-aminomethyl-3 hydroxy 1,4 naphthoquinone compounds could inhibit the early and late phases of HSV-1 replication, after 1 h of viral incubation (MOI of 0.1) at 37 °C, Vero cells were washed three times with MEM, 5% FBS and incubated during 3 h or 6 h. Then, four times the EC_50_ values of each liposome were added to the medium and incubated for an additional 3 h or 14 h, representing, respectively, the early (3–6 h) and late (6–20 h) phases of HSV-1 replication. At the end of incubation, the supernatant was recovered, diluted (1:10) and the percentage of viral inhibition was defined using plaque assay counts, based on the HSV-1 control.

### 4.7. Statistical Analysis

All assays were performed at least three times in triplicate and the statistical analysis was performed using GraphPad Prism 7.0 (GraphPad Software Inc., San Diego, USA). The analysis of variance test was used, followed by multiple comparisons using the Kruskal–Wallis test. Differences were considered statistically significant when *p* < 0.05

## Figures and Tables

**Table 1:** Size (diameter) and zeta potential measurements of neutral Egg-PC liposomes encapsulating the drugs.

Compound	Drug (radical)	Size (nm)	Polydispersity Index	Zeta Potential (mV)
1	n butyl	102.1 ± 1.1	0.19 ± 0.01	−24.2 ± 0.1
2	benzyl	130.1 ± 7.2	0.13 ± 0.09	−20.0 ± 0.1
3	nitrobenzene	112.6 ± 3.5	0.17 ± 0.02	−13.1 ± 0.7

Size, expressed as z-average, and polydispersity index (PDI), are measured by DLS. Data are expressed as means ± standard deviation (SD) of three separate experiments for each of two batch formulations, with at least 13 measurements for each.

**Table 2:** Comparative analysis of CC50 (μM) values of acyclovir and 2-aminomethyl-3-hydroxy1,4 naphthoquinone derivatives, encapsulated and not encapsulated (free compounds), in liposomes performed in Vero cells.

	Acyclovir	1	2	3
encapsulated	13 ± 1	15 ± 1	11 ± 1	13 ± 2
free	15 ± 1	19 ± 1	22 ± 2	17 ± 2

**Table 3:** Values of cell viability (CC50), antiviral activity (EC50) and selective index (SI) of acyclovir and 2-aminomethyl-3-hydroxy-1,4 naphthoquinones derivatives encapsulated in liposomes.

Drug (radical)	CC_50,_ μM	EC_50,_ μM(*)	SI, CC_50_/EC_50_
Acyclovir	13 ± 1	3.16 ± 0.09	4.1
1 (n butyl)	15 ± 1	1.73 ± 0.08	8.7
2 (benzyl)	11 ± 1	0.56 ± 0.02	20
3 (nitrobenzene)	13 ± 2	0.36 ± 0.04	36

(*) EC_50_–drug concentration, which reduced 50% of HSV-1 replication when compared to control. SI represents the ratio between cytotoxicity and the antiviral effect and indicates effectiveness of drugs.

## Data Availability

Not applicable.

## Sample Availability

Samples of the compounds are not available from the authors.
